# Clinical validation of molecular markers of macrocyclic lactone resistance in *Dirofilaria immitis*

**DOI:** 10.1016/j.ijpddr.2018.06.006

**Published:** 2018-07-18

**Authors:** Cristina Ballesteros, Cassan N. Pulaski, Catherine Bourguinat, Kathy Keller, Roger K. Prichard, Timothy G. Geary

**Affiliations:** a Institute of Parasitology, McGill University, 21111 Lakeshore Road, Sainte Anne de Bellevue, H9X 3V9, QC, Canada; b School of Veterinary Medicine, Louisiana State University, Skip Bertman Drive, Baton Rouge, LA, 70803, USA

## Abstract

Prophylaxis with macrocyclic lactone (ML) endectocides is the primary strategy for heartworm control. Recent evidence has confirmed that ML-resistant *Dirofilaria immitis* isolates have evolved. Comparison of genomes of ML-resistant isolates show they are genetically distinct from wild-type populations. Previously, we identified single nucleotide polymorphisms (SNPs) that are correlated with phenotypic ML resistance. Since reliable *in vitro* assays are not available to detect ML resistance in L3 or microfilarial stages, the failure to reduce microfilaraemia in infected dogs treated with an ML has been proposed as a surrogate clinical assay for this purpose. The goal of our study was to validate the genotype-phenotype correlation between SNPs associated with ML resistance and failure to reduce microfilaraemia following ML treatment and to identify a minimal number of SNPs that could be used to confirm ML resistance. In this study, 29 participating veterinary clinics received a total of 148 kits containing supplies for blood collection, dosing and prepaid shipping. Patients recruited after a diagnosis of heartworm infection were treated with a single standard dose of Advantage Multi^®^ and a blood sample taken pre- and approximately 2–4 weeks post-treatment. Each sample was processed by performing a modified Knott's Test followed by isolation of microfilariae, genomic DNA extraction and MiSeq sequencing of regions encompassing 10 SNP sites highly correlated with ML resistance. We observed significant correlation of SNP loci frequencies with the ML microfilaricidal response phenotype. Although all predictive SNP combination models performed well, a 2-SNP model was superior to other models tested. The predictive ability of these markers for ML-resistant heartworms should be further evaluated in clinical and epidemiological contexts.

## Highlights

• Recent evidence has confirmed that heartworm isolates have evolved the ability to survive ML prophylaxis.
• SNPs are highly correlated with phenotypic ML resistance, but none identifies a specific “resistance” gene.
• SNPs associated with ML resistance correlate with failure to reduce MF following ML treatment.
• A 2-SNP model could be employed to diagnose clinical cases of ML resistance.

## Graphical abstract

**Figure:**
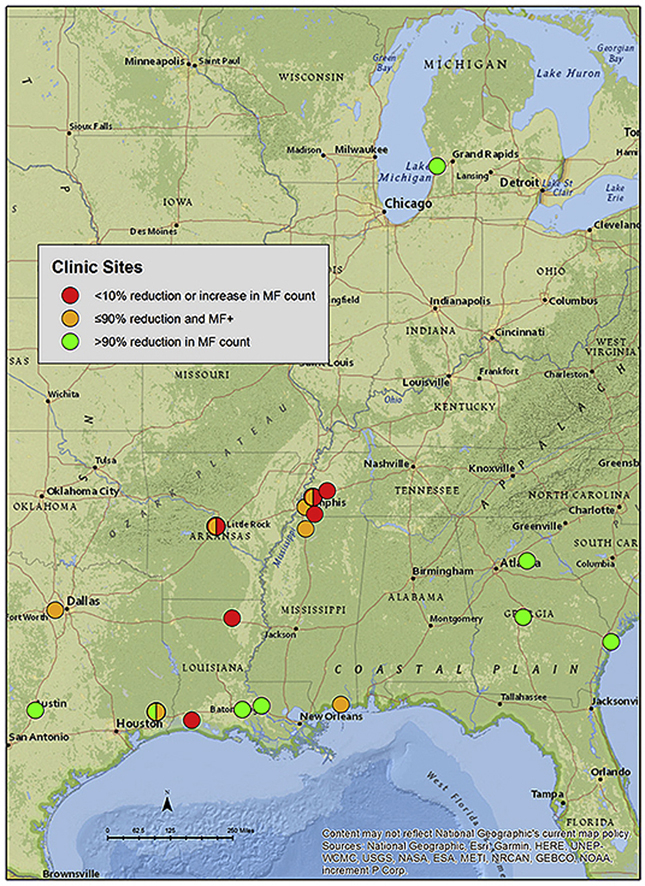

## 1 Introduction

The administration of macrocyclic lactone (ML) preventive drugs is the standard of care for prophylaxis of heartworm (*Dirofilaria immitis*) disease in companion animals, since the introduction of ivermectin (IVM) for this indication in 1987 (Bowman and Atkins, 2009; Geary, 2005; McCall et al., 2008). IVM and subsequently introduced MLs, including milbemycin oxime, moxidectin, and selamectin, provide extremely high efficacy when used properly, and have fewer adverse effects than daily diethylcarbamazine (DEC), the previously approved treatment. However, within about 20 years after the introduction of IVM, concerns about the possible emergence of drug resistance appeared; the Center for Veterinary Medicine, US Food and Drug Administration (CVM/FDA) published a report on the increasing number of lack of efficacy (LOE) claims of MLs to prevent canine heartworm disease (Hampshire, 2005). These LOE reports, wherein animals had diagnosed heartworm infection despite apparent or claimed adherence to accepted guidelines for prophylaxis, had doubled in recent years and were centered in the Lower Mississippi River delta region (Hampshire, 2005; Pulaski et al., 2014). This pattern suggests that ML resistance may have arisen in this area of intense transmission (and related high heartworm disease prevalence rates) of *D. immitis*.

It has proven challenging to determine whether LOE case reports are due to inadequate exposure to MLs, either inadvertently or because of pet-owner drug non-compliance, or to genetically-based resistance. One reason for this difficulty is that MLs have little apparent effect on survival and behaviour of microfilariae (MF) or L3 larvae, the accessible stages of *D. immitis*, in culture at pharmacologically-relevant concentrations (Bourguinat et al., 2011a; Evans et al., 2013; Geary and Moreno, 2012; Moreno et al., 2010; Vatta et al., 2014; Wolstenholme et al., 2015). It has thus been challenging to link a change in clinical ML sensitivity to a reliable and relevant change in parasite survival or behaviour in a laboratory assay. This difficulty is compounded by the fact that the stages of the parasite life-cycle targeted by the MLs, L3 and L4 larvae, are not generally available for routine experimental analysis in most laboratories, as they are difficult to maintain in culture. The ‘gold standard’ test of resistance is to demonstrate that a normally effective ML regimen fails to provide full protection against the development of heartworm disease following experimental infection of laboratory dogs with suspected drug-resistant *D. immitis* isolates. Reports of incomplete ML protection against laboratory strains have appeared (Blagburn et al., 2011; Snyder et al., 2011a, 2011b). More recently, field isolates that broke through ML prophylaxis have been recovered and confirmed following laboratory establishment and experimental animal infection, proving the existence of authentic ML-resistant strains of *D. immitis* in circulation in canine host populations (Bourguinat et al., 2015; Pulaski et al., 2014). However, tests of resistance to prophylaxis utilizing the canine infection model are laborious, time-consuming and expensive, and are not likely to be useful to routinely measure ML resistance status in clinical isolates.

As an alternative, we proposed (Geary et al., 2011) a simple, cost-effective clinical assay as an indicator of ML resistance, based on historical data available on the responses of circulating MF to ML exposure in infected dogs (Bowman, 2012; Bowman and Mannella, 2011). These data demonstrate a precipitous drop in microfilaraemia following the administration of elevated doses of IVM (∼50 μg/kg), even prior to chemotherapeutic removal of adult worms (although microfilaraemia rebounds eventually if adults are not killed). Additionally, one ML, Advantage Multi^®^ (imidacloprid + moxidectin), has a label indication for the treatment of circulating MF, based on laboratory and field studies demonstrating nearly 100% efficacy in eliminating this life-stage in infected dogs (Bowman et al., 2015; McCall et al., 2014a). Therefore, either administration of high-dose IVM or Advantage Multi^®^ should result in a significant decrease (>90%) in the number of circulating MF prior to adulticidal treatment. Although the test does not address the life-cycle stages of the parasite targeted by ML prophylaxis, it can be easily done by quantifying MF loads at two clinic visits (one at the time of IVM or Advantage Multi^®^ treatment, and one 2–4 weeks later, respectively) and, if the mechanism of resistance is conserved and functionally expressed in MF, offers a useful phenotypical marker for ML resistance.

Interrogation of the genomes of suspected ML-resistant strains of *D. immitis* has revealed that these strains are genetically distinct from wild-type strains (Bourguinat et al., 2011b, 2015, 2017b; Geary et al., 2011; McCall et al., 2014b; Pulaski et al., 2014). We have previously identified a number of single nucleotide polymorphisms (SNPs) that are highly correlated with ML resistance as defined by prophylaxis failure (Bourguinat et al., 2015, 2017a); although no individual SNP identifies a single gene that accounts for the mechanism of ML resistance, a set of 4–5 SNPs is highly correlated with the phenotype (Bourguinat et al., 2017a). A diagnostic test based on SNP markers of ML resistance would be of significant value in distinguishing clinical cases of heartworm disease due to ML resistance from those due to drug compliance failure, as well as for monitoring the extent and spread of resistant genotypes and determining the influence of alternative chemotherapeutic strategies, such as the administration of doxycycline (McCall et al., 2014b), on the spread of ML resistance. However, molecular markers require clinical validation to establish a correlation between genotype and phenotype. The purpose of this work was to identify a pattern of SNPs associated with ML resistance, based on the phenotype of MF response to a microfilaricidal ML, which can be used to characterize the resistance status of parasites presenting in infected dogs at the clinic. Improving our ability to distinguish infections that have occurred due to inadequate adherence to a prophylaxis regimen from infections that are caused by a genetically resistant population of heartworms can be of use in educational campaigns to boost client compliance and for tracing the spread of resistant parasites in the field.

## 2 Methods

### 2.1 Preparation of kits

For each dog enrolled in the study, pre- and post-treatment kits were shipped to the participating veterinary clinic. Each Styrofoam shipping kit contained equipment for venipuncture blood collection and shipment, including two 6 ml ETDA blood tubes, blood collection needle and holder, ice packs, and a bio-labelled shipping bag with pre-paid label to McGill University. The pre-treatment kit included one weight-appropriate treatment of Advantage Multi^®^, a topically administered ML (moxidectin), with a FDA label indication for the removal of circulating MF. The post-treatment kit included a brief questionnaire on patient history, including previous ML administration. Responses to these questionnaires have been summarized in S4 Table.

### 2.2 Samples and patient recruitment

Collaborating researchers in various Animal Health companies with an interest in heartworm therapeutics provided contact details for veterinary clinics that indicated a willingness to learn more about the project. Additional potential clinic partners were identified after an appeal for support at the Triennial Meeting of the American Heartworm Society in New Orleans, LA, USA (11–13 September 2016). These clinics were contacted by one of the authors (CNP) to communicate the study goals and protocols, arrange for blood collection kit shipment and return to McGill University, and project follow-up. Veterinarians enrolled clients with the following patient inclusion criteria: dogs ≥1 year of age at the time of examination and in general good health, and capable of donating two 5 ml samples of venous blood approximately 14–28 days apart. Dogs with a newly diagnosed case of patent heartworm infection were selected for the study (based on antigen testing and visual inspection of a blood film to verify the presence of MF). At the initial visit, participating clients authorized the withdrawal of a 5 ml blood sample by venipuncture into an EDTA tube from the dog, followed by the administration of a weight-appropriate dose of Advantage Multi^®^ (generously donated for the study by Bayer Animal Health, Shawnee, KS). This sample was then shipped immediately to McGill University in a pre-paid shipping container, where it was further processed as described below. The client returned to the clinic 2–4 weeks (optimally) later, and a second blood sample was obtained from the dog and immediately shipped to McGill University for processing in the same manner.

### 2.3 Sample processing and DNA extraction

One to 2 tubes of blood samples (5 ml blood per tube) from MF positive dogs were collected in EDTA tubes before and after ML treatment and were immediately shipped to McGill University for processing.

To assess ML phenotypic response, a Modified Knott's Test was performed on 1 ml blood taken from each sample (Mylonakis et al., 2004). A total of 8 counts were performed for each sample and the average was used to calculate the % MF change post-treatment. The remaining blood was used for MF extraction, followed by DNA extraction and genotyping.

MF were extracted and washed from the remaining blood using a filtration procedure (Bourguinat et al., 2015). Polycarbonate membrane filters (3.0 μm; 25 mm; Sterlitech^®^ Corporation, Kent, WA, USA) were used for the filtration. Venous blood was diluted 1:1 with NaHCO_3_ (Sigma-Aldrich, Oakville, ON, Canada) solution (2 g/L) which was prepared before filtration. DNA from pooled MF was extracted using a QIAamp^®^ DNA Micro kit (Qiagen Inc., Toronto, ON, Canada). DNA concentrations were assessed using the Quant-iT™ PicoGreen DNA Assay Kit (Invitrogen^®^, Life Technologies Inc., Burlington, ON, Canada). DNA samples were stored at −80 °C until the end of the sample collection period.

### 2.4 10 SNP markers

Based on previous work, the 10 SNPs that best differentiated the ML-susceptible phenotype from the resistant phenotypes were selected for analysis (Bourguinat et al., 2017a). The list of SNPs evaluated in this study, including what is known or predicted about their location in the genome, are available in S5 Table.

### 2.5 DNA sequencing

Following the sample collection period and processing, DNA samples were sent to the McGill University and Genome Quebec Innovation Centre and regions encompassing the 10 SNPs of interest were sequenced on the Illumina MiSeq Platform, at a coverage of 2000X.

Target enrichment was performed on the Fluidigm Access Array system, which involves an array-based PCR amplification of genomic target regions. Parallel amplification of 48 samples was carried out using custom primers (S5 Table) to which CS1 and CS2 tails were added. Samples were barcoded during target enrichment to allow for multiplexed sequencing and adapter sequences were added during the PCR amplification reaction.

### 2.6 Data analysis

Illumina sequencing adapters were removed from the reads and adapter clipping, trimming for minimal trailing quality (30 PHRED score) and filtering for minimum read length were performed using Trimmomatic (Bolger et al., 2014). Resulting read pairs were then aligned to the *D. immitis* genome reference sequence nDi.2.2 (http://www.nematodes.org/genomes/dirofilaria_immitis). using BWA-mem (http://bio-bwa.sourceforge.net/) (Li and Durbin, 2009) resulting in binary alignment map files (BAM). Alignments where subsequently processed with Picard (https://broadinstitute.github.io/picard) for re-alignment of indels, mate fixing and marking of duplicate reads. BVATools (https://bitbucket.org/mugqic/bvatools/src) was employed to extract base frequencies at each of the 10 SNP positions for each of the BAM files produced. The read frequencies were assimilated to the allele frequencies. The variance of the allele frequency at a given SNP position was compared to previously described allele frequencies for RES and SUS populations (Bourguinat et al., 2015, 2017a) by calculating the Fixation Index (F_ST_) at each of the 10 SNP positions for all samples. An average F_ST_ was then calculated from the 10 SNP-F_ST_ values. F_ST_ is a measure of population differentiation due to genetic structure with values from 0 to 1, whereby a value of 0 implies that two populations are interbreeding freely and a value of 1 implies that all genetic variation is explained by the population structure and that the two populations do not share any genetic diversity (Holsinger and Weir, 2009; Kitada et al., 2007). This method allowed us to compare the F_ST_ values to predict the genotype for that sample. F_ST_s were also calculated between pre- and post-treatment samples at the 10 SNP positions to test for genotypic changes due to ML treatment. This allowed us to assess the possibility of mixed populations present pre-treatment, which would be reflected by significant changes in allele frequencies post-treatment.

### 2.7 Statistical analysis

To identify the best combination of SNP markers that predict ML resistance, the Random Forest algorithm was applied in the “Biomarker analysis” module in MetaboAnalyst 4.0 (http://www.metaboanalyst.ca) (Xia et al., 2012; Xia et al., 2009; Xia et al., 2015; Xia and Wishart, 2011a; b) as previously described (Bourguinat et al., 2017a). Individual SNP marker performance was first identified and sorted in order of highest to lowest AUC. This was followed by manually testing different combinations of SNP markers for ability to predict ML resistance. A score of 0 was the optimal value for ML susceptibility prediction and a score of 1 was the optimal value for ML resistance prediction. A cut-off of 0.5 was employed; a sample with a predicted class probability <0.5 was considered ML susceptible and a sample with a predicted class probability >0.5 was considered ML resistant. For each combination of SNP markers, a ROC curve was built to specify the sensitivity [True Positive/(True Positive + False Negative)] and the specificity [True Negative/(False Positive + True Negative)] of the different SNP combinations. The “Statistical Analysis” module in MetaboAnalyst 4.0 was used to build a heat map using the F_ST_ values calculated for each SNP position when comparing the allele frequencies versus the SUS population allele frequencies for all samples. A second focus heat map was created using the top SNP markers. The Euclidean distance method (Ibaraki, 1986) and Ward clustering algorithm (Ward, 1963) were employed to create the heat maps. The heat maps allowed us to intuitively visualize our dataset. In addition, a PCA 2 Dimension Scores Plot was created using the SNP-F_ST_ data calculated for each sample versus the previously described SUS profile to visualize the data spatially and reveal clusters of groups (i.e., ML-susceptible vs ML-resistant).

## 3 Results

### 3.1 Distribution of samples

**Fig. 1:**
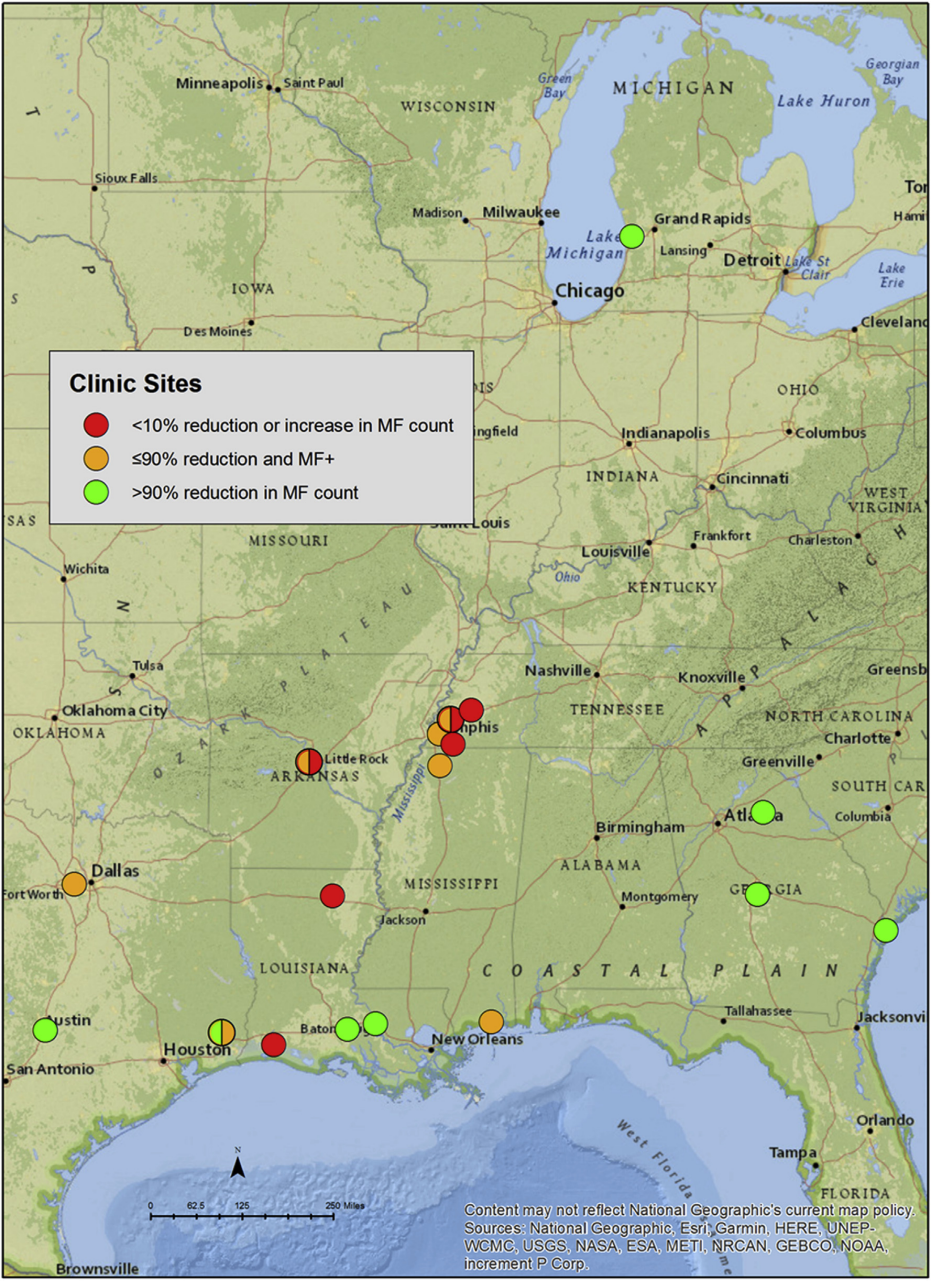
Distribution of samples received. Markers are colour-coded based on the percent change in MF count post-treatment as per the legend. Circles with slit colours indicate there were 2 samples from the same location with different phenotypes based on microfilaria reduction. The map was created using ArcGIS^®^ software (Version 10.5) by Esri (www.esri.com). ArcGIS^®^ and ArcMap™ are the intellectual property of Esri and are used herein under license. Copyright ^©^ Esri. All rights reserved. (For interpretation of the references to colour in this figure legend, the reader is referred to the Web version of this article.)

A total of 148 kits were sent out to 29 participating veterinary clinics and 70 kits were returned. Participating veterinary clinics were geographically spread across the Southeastern part of the USA, mainly around the Mississippi Delta region. Several cases were from Michigan, although two of these dogs were rescue dogs transferred from Tennessee. Fifty samples were submitted for DNA sequencing however one case (samples 24A and 24B) had very low pre and post-treatment MF counts (518 mf/ml and 6 mf/ml respectively) and hence very low DNA concentrations and were not able to be sequenced. Other post-treatment samples whereby MF counts were too low or MF-free were not sent for DNA sequencing. Fig. 1 shows the locations of clinics and types of samples received from each site based on the microfilaria reduction after ML treatment.

### 3.2 ML phenotype response

To assess the phenotypic response to ML treatment, the percentage (%) change in MF counts post-treatment was calculated (Table 1). Samples were grouped into 3 categories: >90% reduction in MF count; ≤90% reduction and MF positive post-treatment; and <10% reduction or increase in MF count post-treatment. Ten cases had >90% reduction in MF count 2–4 weeks post-treatment, 11 cases had ≤90% reduction and were still MF positive post-treatment and 6 cases had <10% reduction or an increase in MF count.

**Table 1:** Sample MF counts before and after ML treatment, calculated F_ST_, and predicted profile.

Sample ID	Clinic Location	MF count pre-treatment (mf/ml)^a^	MF count Post-treatment (mf/ml)^a^	Percentage MF change	F_ST_ (Sample vs SUS profile)^c^	F_ST_ (Sample vs RES profile)^c^	Profile predicted based on genotype and phenotype
>90% Reduction In MF Count							

20A and 20B	Bogart (GA)	14425	0	−100	0.016	0.198	SUS
21A and 21B	Savannah (GA)	2490	0	−100	0.005	0.232	SUS
31A and 31B	Lumberton (TX)	2050	0	−100	0.045	0.125	SUS
37A and 37B	Austin (TX)	6750	0	−100	0.021	0.206	SUS
38A and 38B	Austin (TX)	3263	0	−100	0.024	0.185	SUS
52A and 52B	Breaux Bridge (LA)	650	0	−100	0.271	0.062	MIXED^b^
57A and 57B	Baton Rouge (LA)	363	0	−100	0.157	0.055	MIXED^b^
50A and 50B	Holland (MI)	1188	12.5	−99	0.096	0.060	MIXED^b^
39A and 39B	Warner Robins (GA)	10200	125	−99	0.033	0.169	SUS
40A and 40B	Warner Robins (GA)	4763	138	−97	0.006	0.229	SUS
≤90% Reduction and MF positive post-treatment							
73A and 73B	Little Rock (AR)	17638	1600	−90	0.199	0.015	RES
69A and 69B	Memphis (TN)	19375	2438	−87	0.256	0.046	RES
47A and 47B	Arlington (TX)	2950	488	−83	0.037	0.130	MIXED^b^
76A and 76B	Holland (MI)	9538	1625	−83	0.158	0.090	MIXED^b^
70A and 70B	Memphis (TN)	14938	2625	−82	0.190	0.021	RES
43A and 43B	Coldwater (MS)	65100	19688	−70	0.147	0.021	RES
75A and 75B	Holland (MI)	9838	3113	−68	0.186	0.022	RES
32A and 32B	Lumberton (TX)	1988	663	−67	0.102	0.067	MIXED^b^
63A and 63B	Diberville (MS)	36563	12713	−65	0.056	0.120	MIXED^b^
29A and 29B	Arlington (TX)	2408	1013	−58	0.103	0.067	MIXED^b^
53A and 53B	Brownsville (TN)	1213	600	−51	0.152	0.091	MIXED^b^
<10% Reduction or increase in MF count							
74A and 74B	Little Rock (AR)	26675	24513	−8	0.153	0.018	RES
67A and 67B	Lake Charles (LA)	238	250	5	0.029	0.186	MIXED^b^
14A and 14B	Collierville (TN)	2993	3813	27	0.112	0.021	MIXED^b^
54A and 54B	Brownsville (TN)	313	638	104	0.081	0.098	RES
77A and 77B	Holland (MI)	1938	4150	114	0.173	0.046	RES
6A and 6B	Monroe (LA)	1525	10925	616	0.139	0.021	RES

a Average of 8 counts.

b Mix of susceptible and resistance markers and also based on MF reduction.

c F_ST_ values are based on all 10 markers.

### 3.3 Genotyping of samples and profile prediction

For each sample, pooled MF were subjected to genomic DNA extraction. DNA samples were sent to the Genome Quebec Innovation Centre for analysis, where 10 regions surrounding each SNP marker of interest were sequenced on the MiSeq platform and reads were aligned to the *D. immitis* reference genome. Base frequencies at each of the 10 SNP positions were extracted and compared to previously characterized resistant (RES) and susceptible (SUS) profiles by calculating pairwise SNP-F_STs_ for each position and then calculating the average. The F_STs_, across the 10 markers, for each pre-treatment sample, compared to the RES and SUS profiles, are shown in Table 1. A prediction of the profile (SUS or RES) was based on the % MF reduction and assessment of the genotype based on allele frequencies when compared to the allele frequencies at these SNP positions for the previously characterized profiles. Some samples were termed “MIXED” since they had a mix of SUS and RES markers. For categorical purposes in the Random Forest analysis, which was performed to evaluate the SNP markers as predictors of ML-resistance, samples were categorized as either SUS or RES with a value of 0 or 1 assigned, respectively, and pairwise F_ST_ values when compared to the SUS profile were used for the analysis (S1 Table). Pairwise F_STs_ were also calculated between pre- and post-treatment samples to test for genotypic changes due to ML treatment. Results are included in S2 Datasets. The S2 Datasets also include the read counts and base frequencies extracted from the sequencing data for the 10 SNP positions evaluated for every sample (pre- and post-treatment).

### 3.4 Prediction of optimal SNPs combinations as markers of ML resistance

To predict the best combination of SNPs that differentiate between ML-resistant and -susceptible isolates, based on the MF % reduction following treatment, a ROC (receiver operating characteristic) curve-based model evaluation employing a Random Forest algorithm was implemented in the Biomarker module in MetaboAnalyst 4.0 (http://www.metaboanalyst.ca/). Various combinations of 2, 3, 5, and 10 SNPs were manually selected to generate a series of predictive models. ROC curve analysis is a statistically valid method for biomarker performance evaluation. To compare the performance of different biomarker models, ROC curves can be summarized into a single metric known as the area under the ROC curve (AUC) (Xia et al., 2015). Individual SNP performance evaluations were initially identified and ranked according to the AUC (Table 2) which provided some guidance in selecting the combinations to compare. The program allows the user to select features manually based on their overall ranks (AUC, T-statistic or fold changes) or the user may use K-means (KM) clustering to detect features with similar behaviour to help reduce the redundancy in biomarkers. For this analysis, we used the AUC ranking; therefore, it is important to note that our SNP markers may be the best markers for this particular data set, but not necessarily for different samples.

**Table 2:** Individual SNP marker performance identified with MetaboAnalyst using the Random Forest algorithm.

SNP Position	AUC	T-tests	Fold Change	KM Cluster
nDi.2.2scaf00046_76278	1	0.00003	−4.2739	5
nDi.2.2scaf00046_22857	0.99118	0.00013	−4.455	5
nDi.2.2scaf00046_222254	0.94706	0.00003	−3.8658	1
nDi.2.2scaf00185_10639	0.94118	0.00019	−3.7512	1
nDi.2.2scaf00185_62174	0.93529	0.00002	−3.2358	1
nDi.2.2scaf00140_30919	0.87647	0.00546	−2.9704	2
nDi.2.2scaf00005_662854	0.84706	0.00830	−2.5929	4
nDi.2.2scaf00004_79766	0.75294	0.07718	−0.94211	3
nDi.2.2scaf00001_466197	0.74118	0.01485	−2.243	4
nDi.2.2scaf00587_12915	0.65882	0.14565	−1.0822	3

**Fig. 2:**
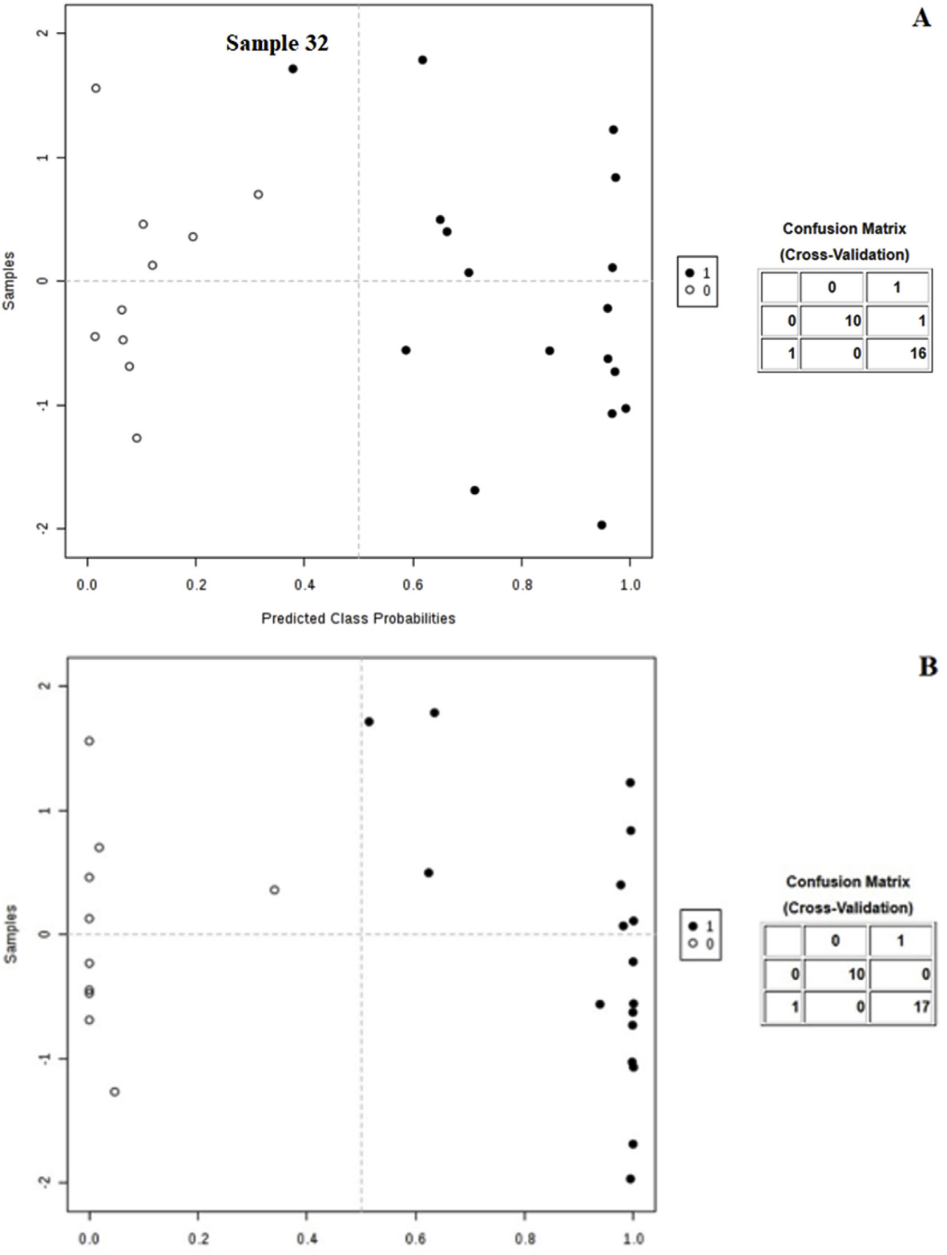
Predicted class probabilities of each sample across 100 cross-validations. (A) shows the predicted class probabilities using the 10 SNP markers and (B) the best 2 SNP markers based on highest AUC values as predictors of ML resistance and performance summary (confusion matrix). Samples predicted in the wrong groups are labeled. The classification boundary is located at the center (x = 0.5). A predicted class probability <0.5 is considered ML-susceptible and a predicted class probability >0.5 is considered ML-resistant.

**Fig. 3:**
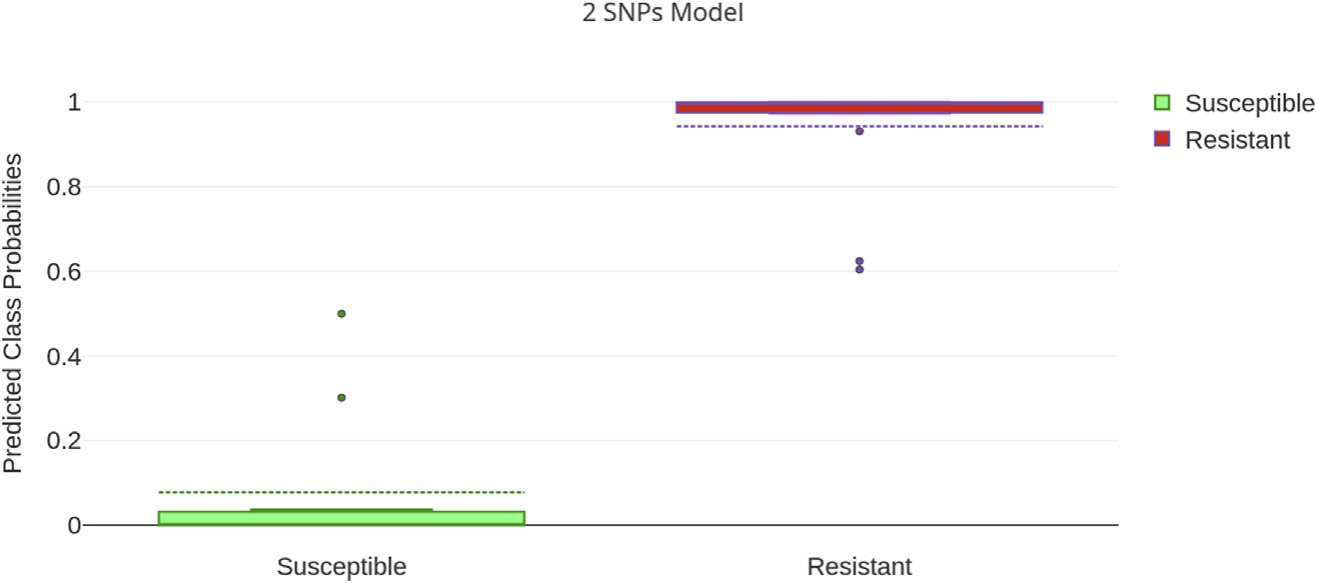
Boxplot of the predicted class probabilities in the 2-SNPs mathematical model. The model was analyzed using the Biomarker module in Metaboanalyst 4.0 with the Random Forest algorithm to predict ML susceptibility or resistance in *Dirofilaria immitis*. Zero was the optimal value for ML susceptibility prediction and 1 was the optimal value for ML resistance prediction. A cut-off at 0.5 was set so that samples with a predicted class probability <0.5 were considered as ML-susceptible and samples with a predicted class probability >0.5 were considered as ML-resistant.

Fig. 2A–B shows the average of predicted class probabilities of each sample across 100 cross-validations using the 10 SNPs model and top 2 SNPs model, respectively (based on individual AUC performances; Table 2). The classification boundary is located at the center (x = 0.5). Sample #32 was classified to the wrong group using the 10 SNP markers as predictors of ML resistance. This sample had low MF counts and a 67% MF reduction post-treatment and was classified as “MIXED” (Table 1). The sensitivity and specificity calculated for this model from the confusion matrix (contingency table with two dimensions (“actual” and “predicted”) is 94.1% and 100%, respectively. However, using the top 2 SNP markers, none of the samples were classified incorrectly in the predicted groups (Fig. 3). The sensitivity and specificity calculated from the confusion matrix is 100% for both. The predicted class probabilities for the top 2-SNP markers model is also presented in boxplot format in Fig. 3. Other combinations of 3, 5, and 10 SNPs which were manually selected to generate a series of predictive models are presented in boxplot format in S3 Figure.

Although all predictive models were well supported, a 2-SNP model based on the individual performances in the ROC analysis and the sensitivity and specificity values calculated from the confusion matrix gave the best results overall (Table 3).

**Table 3:** Overall performance of 2, 3, 5 and 10 SNP Predictive Model.

Predictive Model	AUC	Sensitivity (%)	Specificity (%)	Samples classified to wrong group
2 SNP	1	100	100	–
3 SNP	1	94.1	100	#32
5 SNP	0.998	94.1	100	#32
10 SNP	0.983	94.1	100	#32

### 3.5 Data visualization

**Fig. 4:**
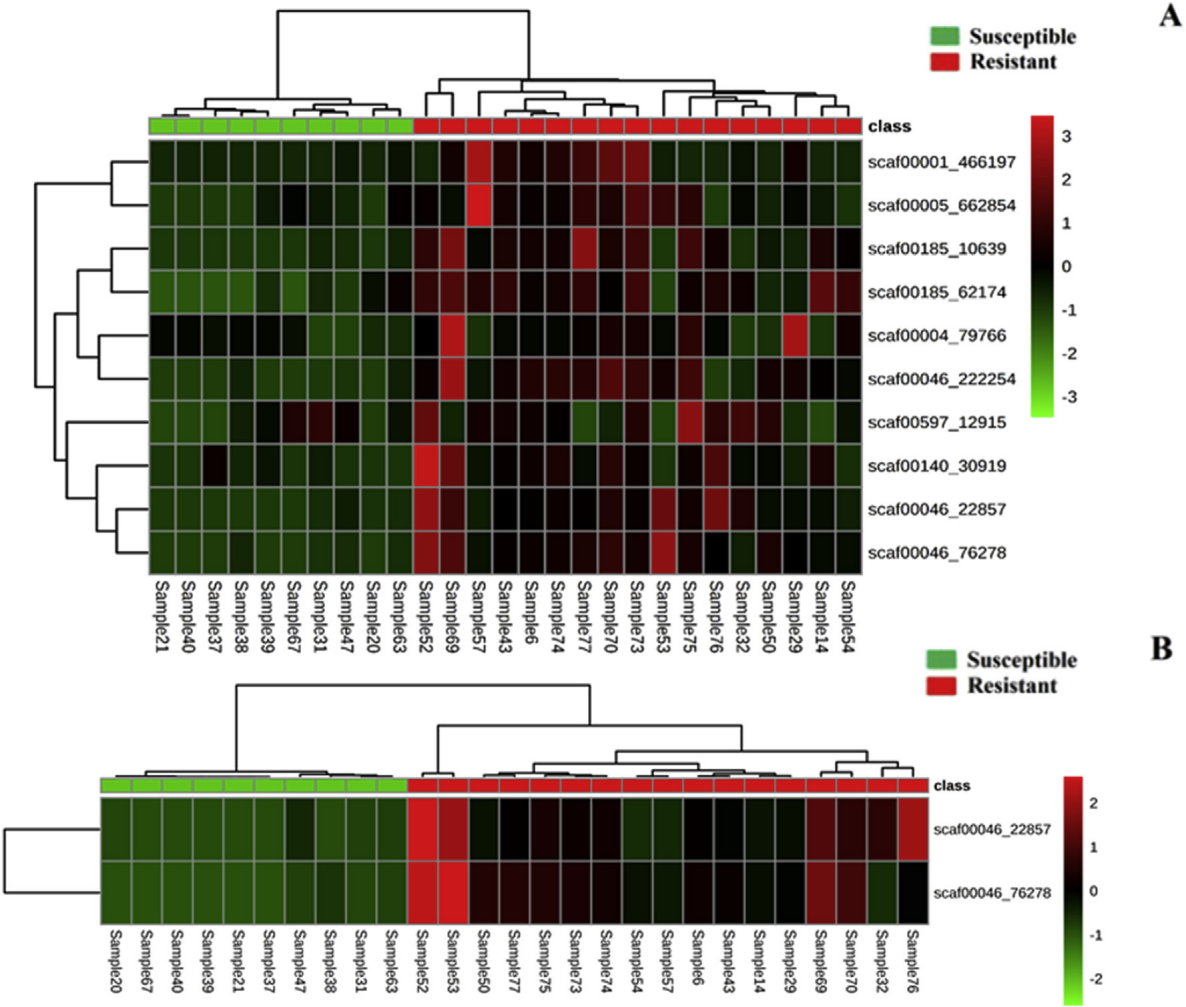
Heat map of pairwise F_ST_ values calculated for each SNP position. (A) All samples versus the SUS population previously described, for all 10 markers, and (B) The best 2 SNP markers based on ROC analysis. The Euclidean distance method and Ward clustering algorithm were both employed to create the heat map in MetaboAnalyst 4.0. The color scheme ranges from −2 (bright green) to 2 (bright red); lower values indicate fewer genetic differences compared to the SUS profile. (For interpretation of the references to colour in this figure legend, the reader is referred to the Web version of this article.)

To visualize our dataset, we used PCA (principal component analysis) and heat map clustering. Both analyses were performed using the Statistics module in MetaboAnalyst 4.0. These methods can be used to emphasize variation and detect outliers and illuminate strong patterns in a dataset. The heat map presented in Fig. 4A allowed us to visualize the predicted phenotype based on the ML response for each sample to be mapped against the F_STs_ calculated at each of the 10 different SNP positions for all samples. In the heat map, the column dendrogram shows two distinct clusters representing samples predicted to be susceptible (green boxes) and samples predicted to be resistant (red boxes). Among the resistant samples, 3 further distinct clusters were apparent. The row dendrogram on the side shows that some of the SNPs from the same scaffolds clustered, such as the 2 bottom rows located on Scaffold 46 (positions 22857 and 76278) and the 2 SNP markers located on Scaffold 185 (positions 10639 and 62174). Scaffolds are defined as overlapping contigs or continuous (not contiguous) sequences resulting from the reassembly of the small DNA fragments separated by gaps of known length (Staden, 1979). In the second heat map (Fig. 4B), we focus on the 2 best SNP markers (both located on Scaffold 46). The heat map shows a clear distinction between predicted susceptible and resistant isolates based on the pairwise F_ST_ values calculated between the samples and the previously described SUS profile. Here we see 2 different clusters among the classified resistant samples; one of the clusters is further broken down into 3 groups (samples 52 and 53; samples 50 to 29; and samples 69 to 76).

**Fig. 5:**
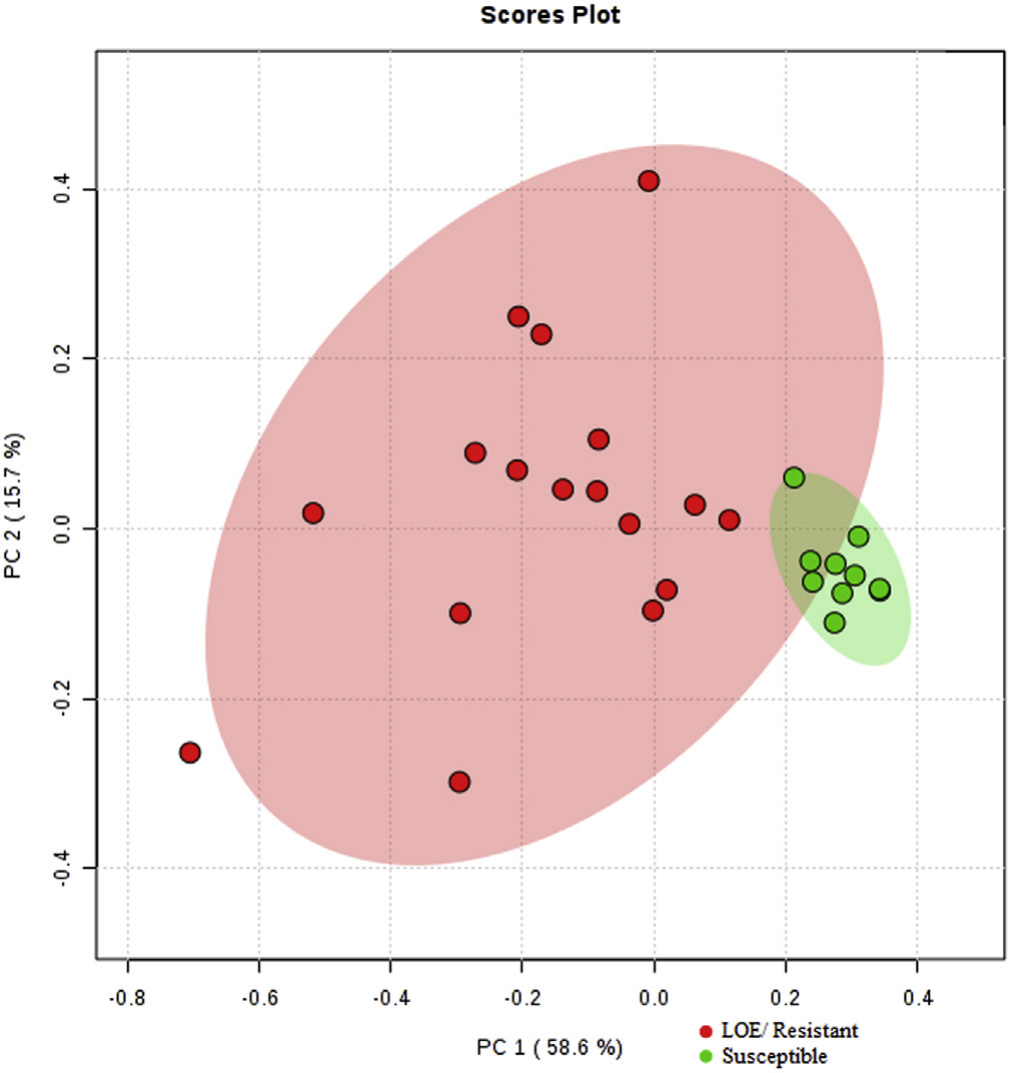
Two-Dimensional Scores Plot created from F_ST_ data (samples versus SUS profile). The shaded areas show the 95% confidence regions. The plot shows two distinct groups whereby red dots represent predicted resistant isolates and green dots represent predicted susceptible isolates. The distance between each dot is a reflection of the genetic differences between each isolate based on the overall average pairwise F_ST_ values for the 10 SNPs compared to the previously characterized SUS profile. (For interpretation of the references to colour in this figure legend, the reader is referred to the Web version of this article.)

Principal component analysis (PCA) can be used to transform original data into a new system of orthogonal axes (components) with the first components covering the major variance in the data (Kastenmuller et al., 2011). Therefore, projections onto the first principal components can often reveal characteristic groups in the data. A 2-Dimensional Scores Plot (Fig. 5) was created using the pairwise SNP-F_ST_ data calculated for each sample versus the previously described SUS profile. The plot shows 2 distinct groups in which samples predicted to be ML-susceptible are highly clustered (green dots) and samples predicted to be ML-resistant (red dots) are distinctly clustered from the susceptible samples yet are spatially spread out. This reflects the range of quantitative ML phenotypic responses across the samples, which is also evident in the heat map. The shaded regions represent 95% confidence regions. As the PCA plot only represents the F_ST_ data and does not reflect the ML-phenotypic response, groupings of samples in the PCA plot were verified against the phenotypes previously predicted and were found to group accordingly with high correlation.

### 3.6 Changes in MF populations before and after treatment

To assess if pre- and post-treatment MF populations were genetically different, we calculated pairwise SNP-F_ST_ values at the 10 positions to examine changes before and after treatment. With the exception of one sample (#32), all pairwise F_ST_ values for each of the 10 SNP positions were close to 0, indicating no differences in the populations. For sample #32, the F_ST_ value at SNP position nDi.2.2. scaf00046: 22857 was 0.28, indicating a change in the allele frequencies at this position post-treatment.

## 4 Discussion

ML-resistant *D. immitis* populations are circulating in dogs in the United States (Bourguinat et al., 2015, 2017a; Pulaski et al., 2014; Wolstenholme et al., 2015). The 2016 heartworm incidence survey by the American Heartworm Society (AHS) reported a 21.7% increase in the average number of dogs diagnosed positive for adult heartworm per clinic (https://heartwormsociety.org/newsroom/in-the-news/347-ahs-announces-findings-of-new-heartworm-incidence-survey), with the Companion Animal Parasite Council (CAPC) reporting a similar increase (15.28% from 2013 to 2016) (Drake and Wiseman, 2018). National ML drug dispensing data analysed from the same time period revealed that the overall proportion of dogs receiving heartworm prophylaxis remained stagnant, with approximately 2/3 of dogs in the United States receiving no heartworm prevention each year (Drake and Wiseman, 2018). Much remains unknown about the distribution of ML-resistant heartworm populations and the extent to which the efficacy of ML endectocides for the prevention of heartworm infection is threatened. Reliable *in vitro* assays are not available to detect ML resistance in L3 or microfilarial stages. A diagnostic test based on SNP markers of ML resistance could help distinguish clinical cases of heartworm infection due to ML resistance from those due to failure of adequate compliance and for monitoring the extent and spread of resistant isolates in epidemiological surveys. Such a test could also determine the influence of alternative chemotherapeutic strategies on the spread of ML resistance, such as the deployment of doxycycline or melarsomine dihydrochloride (Bowman and Drake, 2017; McCall et al., 2014b). Previously, we identified a set of 10 SNPs that were highly predictive of ML resistance (Bourguinat et al., 2017a). In this study, we aimed to clinically validate these molecular markers and establish a correlation between the genotype and phenotype (failure to reduce MF following ML treatment). We also aimed to identify the minimal number of SNP markers that could be used for ML resistance screening in the future.

We used Advantage^®^ Multi (active ingredient: moxidectin), the only ML licensed in the USA as a microfilaricide for dogs, to characterize the phenotype. Previous studies with Advantage^®^ Multi as a microfilaricide in laboratory and field trials (Bowman et al., 2015; McCall et al., 2014a) reported efficacies of essentially 100% in all treated animals 2–6 weeks following dosing. We found that more than half the dogs had responses much lower than this, with five dogs experiencing an increase in microfilaraemia 2–4 weeks following dosing. As the Advantage^®^ Multi was administered in the clinic, failure of compliance is an unlikely explanation for these results.

Based on the % MF reduction post-treatment and assessment of allele frequencies compared to previously characterized SUS and RES base frequencies at the 10 SNP positions, cases were characterized as either ML-susceptible or -resistant. In certain cases, the MF counts were so low that it was challenging to predict the profile based only on the ML phenotypic response. It's important to also note that daily variations can occur in MF counts and the circadian cycle of *D. immitis* MF in the peripheral blood varies considerably between dogs and season (Ionica et al., 2017; Lovis et al., 2017). Nonetheless, we were able to reasonably categorize the phenotypic response of the samples.

Using the predicted profiles for each sample and the pairwise F_ST_ values calculated at each of the 10 SNP positions for all samples versus the SUS profile, a ROC curve analysis was performed and individual SNP marker performance was evaluated. This allowed us to manually select different combinations of SNP markers to test as predictors of ML resistance, including the 10 markers as a set. All models tested very well, with sensitivity and specificity values of 94.1% and 100% for the 10 SNP panel as calculated from the confusion matrix in the predictive class probabilities graph. One sample (#32) was incorrectly classified as RES based on the average F_ST_ values for the 10 markers. The best model was a 2-SNP model based on the top 2 markers in terms of individual ROC curve performance. Sensitivity and specificity for this model was 100% and none of the samples were incorrectly classified based on predictions of the sample profiles. This was also reflected in the focused heat map created using the 2 top markers versus the 10 markers, in which clustering of the SUS and RES samples was well defined due to significant differences between F_ST_ values for these SNPs between both populations. It is interesting to note that the 3 best SNPs in terms of individual performance are located on Scaffold 46. Again, it is important to note that the selection of features based on the overall AUC rank can lead to overfitting and therefore, although these SNP markers performed the best for our dataset, this may not be the case for new samples.

Lastly, to assess if pre- and post-treatment MF populations were different, pairwise SNP-F_ST_ values at the 10 positions were calculated using the allele frequencies before and after treatment. With the exception of one SNP position in sample #32, no statistical differences between the populations before and after treatment were detected, as all SNP-F_ST_ values were close to 0. Case #32 was a stray male dog from Lumberton, Texas, who had a MF count of 1988 mf/ml pre-treatment and 663 mf/ml 3 weeks post-treatment and had not been on heartworm preventatives in the 24 months prior. For cases which we predicted to be RES, we expected to see significant differences in the allele frequencies pre- and post-treatment, suggesting the possibility that the dog harbored both SUS and RES populations before treatment, but this was not the case. Although the mechanism(s) of ML resistance remain to be defined, previous studies reported that ML resistance may be polygenic (Bourguinat et al., 2016, 2017a; McCavera et al., 2007; Prichard, 2001). In this study, we saw a continuous variation in phenotypes and in the pairwise F_ST_ values, observations supporting the hypothesis that ML-resistance is a polygenic trait that may be controlled by multiple genes involved producing a spectrum of phenotypes with different absolute levels of susceptibility to MLs. Therefore, the apparent genetic diversity of the resistant parasites compared to the lack of diversity of the susceptible ones was not surprising, keeping in mind that we focused on 10 SNP markers which were previously shown to be highly correlated with resistance. We expected that there would not be significant differences in allele frequencies at these markers among identified SUS isolates, whereas the allele frequencies at these SNP sites in RES isolates would differ significantly from SUS isolates. This is reflected both in the heat map and the Two-Dimensional Scores Plot analysis. The implications for a multigenic phenotype with varying levels of resistance for the efficacy of different ML products remain to be investigated.

It is important to note some potential sources of bias and limitations in our study. It was a challenge to collect a large number of samples due to the difficulty in enrolling a high number of veterinary clinics willing to participate in the study and recruit patients. In some cases, patients were recruited and received a treatment yet did not return for the post-treatment blood collection so these samples had to be excluded from the study. A higher number of samples could have increased the robustness of our study. Despite some of these drawbacks, we believe we obtained a good geographical distribution of samples. It should also be noted that the results of our study are limited to the use of Advantage Multi (active ingredient: moxidectin), the only ML licensed in the USA as a microfilaricide for dogs. Previously it was shown that the combination of imidacloprid/moxidectin was 100% effective in preventing the development of the JYD-34 laboratory strain of *D. immitis* in dogs following a single preventive treatment, while three monthly treatments of three other heartworm preventive products, consisting of either ivermectin/pyrantel pamoate chewable tablets, milbemycin oxime/spinosad tablets, or selamectin topical solution provided less than 100% preventive efficacy (Blagburn et al., 2016). In the present study, Advantage Multi was assessed for its microfilaricidal activity rather than its heartworm preventive activity, as in the Blagburn et al. study. While we have assessed the microfilaricidal activity of Advantage Multi as a surrogate *in vivo* assay to test for ML resistance, the possibility exists that some of the isolates in our study identified as either SUS or MIXED, based on the MF reduction phenotype, could be resistant to other MLs when used as preventives.

## 5 Conclusion

Our data reveal a strong genotype-phenotype correlation between SNPs associated with ML resistance and failure to reduce microfilaraemia following ML treatment. The correlation was not as significant in cases with low MF counts, emphasizing the value of a diagnostic test for resistant cases based on SNP markers. Random Forest analysis revealed that the set of 10 SNP markers accurately predicted ML-resistance. Although all models performed well, a 2-SNP model performed statistically better than other models tested. A more robust performance estimate could be obtained using more samples. It would also be desirable to repeat this study on a much wider scale geographically using the 2-SNP markers as predictors of ML resistance. Development and adoption of a convenient, robust assay that could be used to sample clinical samples or pools of mosquitos would provide great value for defining the extent of ML resistance in this species and in enabling experimental analysis of variables that influence its spread. Our results suggest that ML resistance is a polygenic trait based on the quantitative variation in phenotypic response in the presence of conserved allelic composition in samples obtained pre- and post-treatment. Lastly, the previous and present work we have undertaken to identify SNPs highly correlated with resistance could provide an excellent model for making similar assessments in human filarial infections. Repeated IVM mass drug administration (MDA) has been ongoing for more than 30 years in endemic countries where filarial diseases such as Lymphatic Filariasis and Onchocerciasis continue to persist (WHO, 2014a; b). In some cases, sub-optimal responses to IVM and genetic changes have been reported suggesting that IVM resistance may already be occurring (Doyle et al., 2017; Nana-Djeunga et al., 2012; Osei-Atweneboana et al., 2007, 2011).

## List of abbreviations

**AUC**: Area under Curve
**CVM**: Center for Veterinary Medicine
**DEC**: Diethylcarbamazine
**DNA**: Deoxyribonucleic acid
**FDA**: Food and Drug Administration
**IVM**: Ivermectin
**KM**: K-Means
**LOE**: Loss of Efficacy
**MDA**: Mass Drug Administration
**MF**: Microfilaria
**ML**: Macrocyclic Lactone
**PCA**: Principle Component Analysis
**RES**: Resistant
**ROC**: Receiver Operating Characteristic
**SNPs**: Single Nucleotide Polymorphisms
**SUS**: Susceptible
