# Supplementary material for: Clinical validation of molecular markers of macrocyclic lactone resistance in Dirofilaria immitis
Source: Int J Parasitol Drugs Drug Resist. 2018 Jul 18;8(3):596–606. doi: 10.1016/j.ijpddr.2018.06.006 (PMC6288007; doi:10.1016/j.ijpddr.2018.06.006)
Supplement: Fig._S3 [file mmc1.pdf]

Supporting File S3. **Mathematical models using the biomarker module in Metaboanalyst 3.0 with Random Forest algorithm to predict macrocyclic lactone susceptibility or resistance in *Dirofilaria immitis*.** Predicted class probabilities for five models are presented here in box plot format based on combinations of 2, 3, 5, or 10 SNPs (n=27 samples with 11 predicted susceptible and 16 predicted resistant, based on microfilariae count reduction following treatment). SNP combinations used for each model tested are shown in the gray shaded boxes. Zero was the optimal value for macrocyclic lactone susceptibility prediction and one was the optimal value for macrocyclic lactone resistance prediction. A cut-off at 0.5 was set, whereby any samples with a predicted class probability less than 0.5 was considered as macrocyclic lactone susceptible and any samples with a predicted class probability greater than 0.5 was considered as macrocyclic lactone resistant.

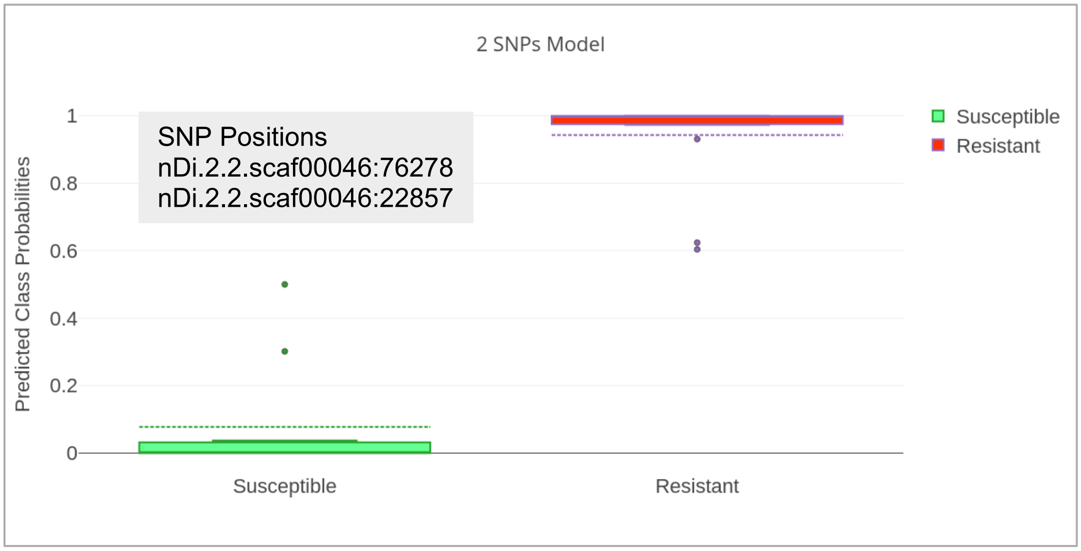

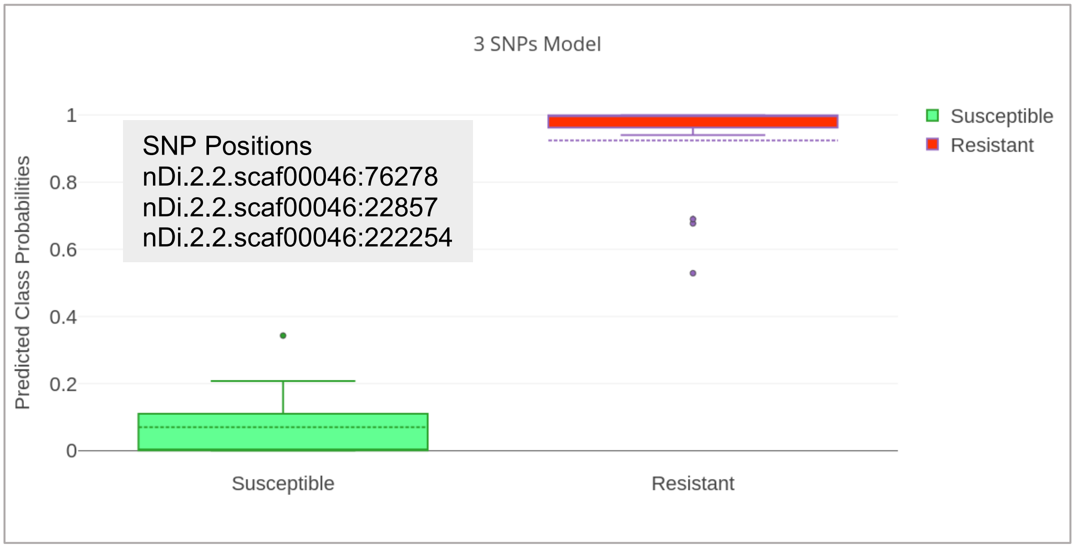

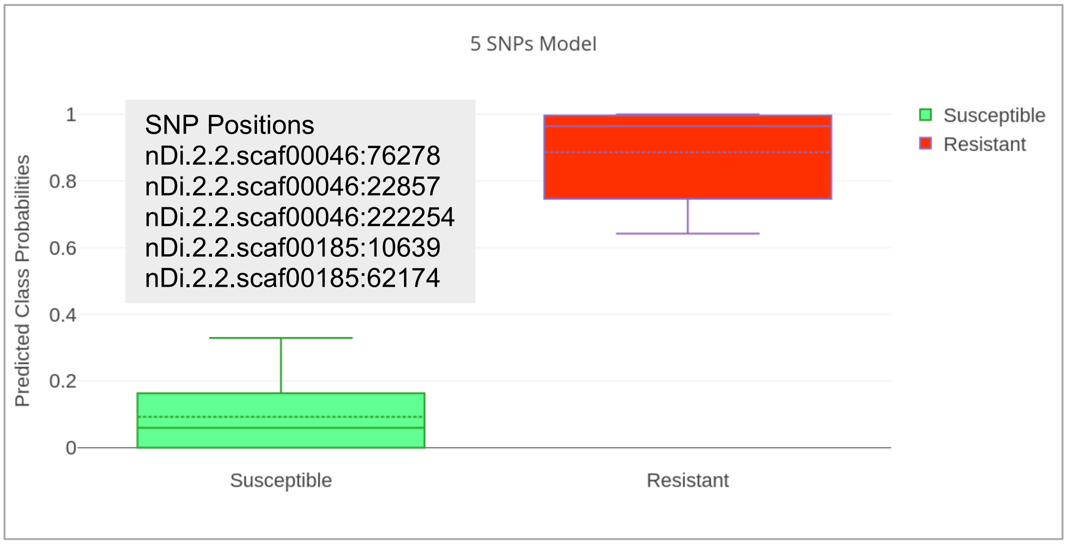

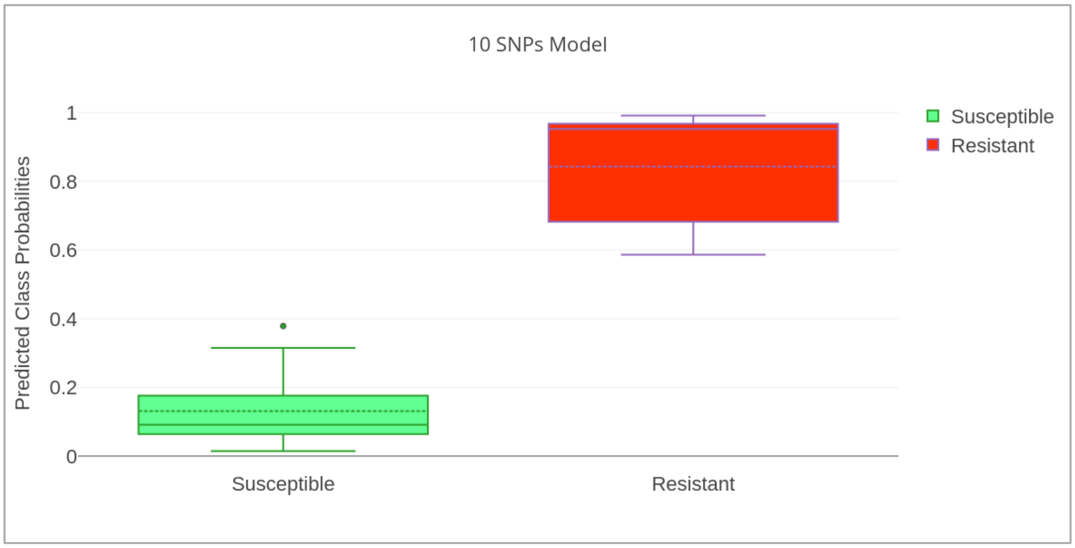

SNP Positions

nDi.2.2.scaf00046:76278	nDi.2.2.scaf00140:30919
nDi.2.2.scaf00046:22857	nDi.2.2.scaf00005:662854
nDi.2.2.scaf00046:222254	nDi.2.2.scaf00004:79766
nDi.2.2.scaf00185:10639	nDi.2.2.scaf00001:466197
nDi.2.2.scaf00185:62174	nDi.2.2.scaf00597:12915
