# Supplementary material for: Clinical validation of molecular markers of macrocyclic lactone resistance in Dirofilaria immitis
Source: Int J Parasitol Drugs Drug Resist. 2018 Jul 18;8(3):596–606. doi: 10.1016/j.ijpddr.2018.06.006 (PMC6288007; doi:10.1016/j.ijpddr.2018.06.006)
Supplement: Table S5 [file mmc2.pdf]

Supporting File S5. SNP Positions and Primer Sequences.

SNP Position and Primer name	Primer Sequence
scaf00001-466197-CS1F	ACACTGACGACATGGTTCTACATTCTATCGAAAACCTTCCAG
scaf00001-466197-CS2R	TACGGTAGCAGAGACTTGGTCTAGGTTGCAAAAGTTGCAATG
scaf00005-662854-CS1F	ACACTGACGACATGGTTCTACAGTTATTTGCACTACTCTCCC
scaf00005-662854-CS2R	TACGGTAGCAGAGACTTGGTCTTGGCGTACTGATCACATTGG
scaf00046-222254-CS1F	ACACTGACGACATGGTTCTACACATCGTTGTCAACTTCCTGC
scaf00046-222254-CS2R	TACGGTAGCAGAGACTTGGTCTGAAATTTGAAAATGGGTACT
scaf00185-10639-CS1F	ACACTGACGACATGGTTCTACAACGCAGGAAAGCTTTAATGG
scaf00185-10639-CS2R	TACGGTAGCAGAGACTTGGTCTATCATCATTTTATCAATTCC
scaf00185-62174-CS1F	ACACTGACGACATGGTTCTACATCGATCATTTAGTAACAACG
scaf00185-62174-CS2R	TACGGTAGCAGAGACTTGGTCTTTGCGTTACAGCGCCAAATC
scaf00004-79766-CS1F	ACACTGACGACATGGTTCTACACGTGACTAAAAGAATAGTG
scaf00004-79766-CS2R	TACGGTAGCAGAGACTTGGTCTCAATTTAGGGATATGACACAG
scaf00046-22857-CS1F	ACACTGACGACATGGTTCTACACGAGGTAAAGCACACAGAAG
scaf00046-22857-CS2R	TACGGTAGCAGAGACTTGGTCTCAACAAAATGCCGCAGATGG
scaf00046-76278-CS1F	ACACTGACGACATGGTTCTACAGGCCAATAAATAAAGGCTA
scaf00046-76278-CS2R	TACGGTAGCAGAGACTTGGTCTGTTTTCTGGAATTATCAGAC
scaf00140-30919-CS1F	ACACTGACGACATGGTTCTACACGAAGAAGAAACTTTTCGGG
scaf00140-30919-CS2R	TACGGTAGCAGAGACTTGGTCTGTACAATTAATTGCTGTTCGC
scaf00597-12915-CS1F	ACACTGACGACATGGTTCTACACGCTTGTTGATTACGACGC
scaf00597-12915-CS2R	TACGGTAGCAGAGACTTGGTCTATGGGGAATTATTACGTTGG
